# Urban Gardening: Managing the Risks of Contaminated Soil

**DOI:** 10.1289/ehp.121-A326

**Published:** 2013-12-01

**Authors:** Rebecca Kessler

**Affiliations:** **Rebecca Kessler** is a science and environmental journalist based in Providence, RI.

Author Rebecca Kessler is all too familiar with the difficulties and uncertainties of cleaning up dirty urban soil, having embarked on a multiyear project to convert a paved parking lot at her Providence, Rhode Island, home into a beautiful and fruitful garden.

*On* a bright late-September afternoon, Mary Bleach showed visitors around the community garden near her apartment in Boston’s Dorchester neighborhood. The sunflowers were nodding their heads in acquiescence to fall, but rust-colored marigolds, pink cosmos, and fuchsia morning glories were still abloom, and a few lazy bees hit them up for nectar. Kale, collards, okra, callaloo (a relative of spinach), tomatoes, onions, herbs, eggplants, beans, peanut plants, and a squash vine with leaves bigger than Bleach’s head entangling 15 feet of chain-link fence—all were still soaking up the fall sun’s rays. Bleach said she lives out of the garden in summer, and she freezes enough to eat well into winter, too.

All this vegetable profusion would soon be gone. Winter was coming, yes, but also heavy machinery to scrape the land level and to haul away the ramshackle chain-link fence and the timbers dividing one plot from another. After more than 25 years, the garden at the corner of Lucerne and Balsam streets was slated for a makeover: handicapped-accessible concrete paths, sturdy fencing, new water service, and reestablished plots with granite dividers.

Boston University toxicologist Wendy Heiger-Bernays and three students had come to check out the site in preparation for a detailed soil contaminant study that would inform the renovation. If the garden’s soil were anything like other Boston soils, it would contain elevated levels of lead—in Dorchester yards, 1,500 ppm of lead is common.^1^ In the worst-case scenario, much of the garden’s soil would have to be removed and clean topsoil and compost trucked in.

And those old timber plot dividers? They were pressure-treated lumber of a vintage that was preserved using chromated copper arsenate—although when they were installed, they were considered a safe alternative to creosote-soaked railroad ties, another common landscaping material. In a 2009 study of three other Boston community gardens, Heiger-Bernays and colleagues showed that arsenic can leach from pressure-treated lumber into garden soil, and that polycyclic aromatic hydrocarbons (PAHs) can leach from old railroad ties.^2^

Heiger-Bernays and her students eyeballed the garden’s perimeter. The adjacent houses were Boston’s signature triple-deckers, probably around a century old and layered in old lead-based paint. Long ago, similar houses stood where the garden now grew. Lead-based paint, asbestos, coal ash, and automotive oil from them could still haunt the garden soil. The lot had stood weedy and trash-strewn for years before Bleach and other neighbors reclaimed it in the 1980s.

The students bagged soil samples near the timbers, along the fenceline adjacent to the houses, and in plots throughout the garden. They would take these samples back to Heiger-Bernays’s lab for analysis.

Over the years the garden has been tested for lead and some clean soil brought in. Recently, the city has brought in truckloads of municipal compost almost every year. This black gold not only supplies nutrients to crops, but also dilutes contaminants and binds them to soil particles, reducing the risk of human exposure.^3^^,^^4^

Over the past decade, the garden’s owner, Boston Natural Areas Network, has systematically renovated select community gardens to further improve and remediate soil as well as to enhance the gardens’ beauty, accessibility, and permanence with high-quality infrastructure. It’s an effort to make growing food in what Heiger-Bernays calls “non-pristine” city soils as safe as possible, so that the many delights of gardening can flourish in the heart of the concrete jungle. “It’s about trying to really maximize those benefits while recognizing and minimizing the risks,” says Heiger-Bernays.

Boston is not alone in its efforts. In cities around the globe, gardeners and farmers are digging into backyards and vacant lots, replacing blighted eyesores with lush, productive vegetation. But as in Boston, these other urban soils are often heavily contaminated, prompting questions about potential health consequences of this supposedly wholesome activity. And while alternative growing methods such as rooftop gardens and hydroponics duck soil contamination issues, they tend to be more expensive and are unlikely to replace gardening in the ground any time soon, sources say.

In the United States, no regulations specifically govern contaminants in soils used for food production, and testing for them can be prohibitively expensive. Experts disagree on the severity of the problem, jurisdictional standards conflict, and advice about how to remedy or work around urban soils has been fragmented and all too often confusing. But recent interest in urban agriculture as a way to green cities, grow jobs, and help quench urban food deserts is bringing new urgency to the research—and a few new solutions.

## Measuring Soil Health

City gardens were not unusual during early U.S. history, but after World War II they largely disappeared. A gardening revival took root amid the urban decay of the middle and late twentieth century. Although data capturing the trend are elusive, food gardening in general is increasing.^2^ In 2012, 35% of U.S. households grew food, spending $3.3 billion in the process, up from 31% of households spending $2.5 billion in 2008, according to the National Gardening Association.^5^^,^^6^ One million households participated in community gardens in 2008, according to the association’s most recent estimate.^7^

An awareness that urban gardeners may be digging into some pretty nasty soil emerged along with the community garden movement in the late 1970s.^8^^,^^9^ A 1983 study identified elevated levels of lead, cadmium, copper, nickel, and zinc in Baltimore inner-city garden soils.^10^ While some common contaminants occur naturally in soil, the levels “were just so high compared to soils found in agricultural areas that it became very clear that these were problematic soils,” says Howard Mielke, a research professor at Tulane University School of Medicine who led the study.

Other studies followed, finding heavily contaminated urban yards and gardens across the United States.^1^^,^^11^^,^^12^ Contaminants tend to concentrate in low-income neighborhoods with large minority populations—although rural areas are not immune.^12^^,^^13^^,^^14^

Lead from old vehicle exhaust, paint, and past industrial activities is the most widely documented pollutant in urban soils. The U.S. Environmental Protection Agency (EPA) estimates that 23% of privately owned U.S. homes built before 1980 have soil lead levels exceeding 400 ppm—the current hazard standard for bare soil in children’s play areas—and that 8% exceed 2,000 ppm.^15^ PAHs, emitted when carbon-containing materials such as wood and gasoline are incompletely burned, are also quite common.

Often a site’s history provides a clue to the contaminants that linger in the soil. Former parking lots and car washes often carry metals, PAHs, petroleum products, solvents, or surfactants. Demolished commercial or industrial buildings may leave behind asbestos, polychlorinated biphenyls, petroleum products, or lead-based paint chips, dust, or debris. High-traffic roadways have a legacy of lead and PAHs from vehicle exhaust. Former parks and lands adjacent to railroad rights-of-way can bear pesticide residues.^4^

Gardeners themselves sometimes introduce potentially dangerous chemicals. Heiger-Bernays is looking into accounts of rising pesticide use in some Boston community gardens, including the use of restricted chemicals, in spite of rules prohibiting them. Biochar—partially burned organic matter, such as charcoal—is another potentially problematic additive. It’s an ancient soil amendment now being touted as a way to combat climate change by sequestering carbon underground.^16^ Yet it’s chock-full of PAHs, Heiger-Bernays points out, some of which may remain more bioavailable than others.^17^

**Figure:**
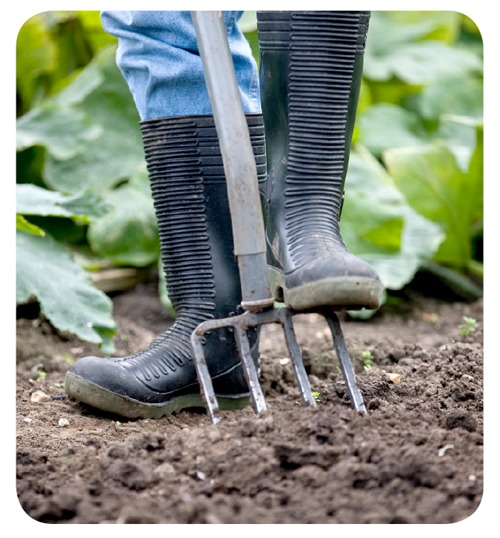
BEST MANAGEMENT PRACTICES FOR URBAN GARDENS Build your garden away from existing roads and railways, or build a hedge or fence to reduce windblown contamination from mobile sources and busy streets. Cover existing soil and walkways with mulch, landscape fabric, stones, or bricks. Use mulch in your garden beds to reduce dust and soil splash, reduce weed establishment, regulate soil temperature and moisture, and add organic matter. Use soil amendments to maintain neutral pH, add organic matter, and improve soil structure. Add topsoil or clean fill from certified soil sources. Your state or local environmental program, extension service, or nursery may be able to recommended safe sources for soil and fill. Build raised beds or container gardens. Raised beds can be made by simply mounding soil into windrows or by building containers. Sided beds can be made from wood, synthetic wood, stone, concrete block, brick, or naturally rot-resistant woods such as cedar and redwood. Your state or local city agency may recommend using a water-permeable fabric cover or geotextile as the bottom layer of your raised beds to further reduce exposure to soils of concern. **Practice good habits:** Wear gloves, and wash hands after gardening and before eating.Take care not to track dirt from the garden into the house.Wash produce before storing or eating, and teach kids to do so, too.Peel root crops, and remove outer leaves of leafy vegetables. Adapted from: U.S. EPA (2011)^4^ Photo: © I Love Images/Corbis

Mielke and his colleagues recently created a detailed map of soil lead and children’s blood lead concentrations across the city of New Orleans, highlighting a strong association between the two.^13^ Mielke says similar studies could and should be done nationally for a host of contaminants. “It’s amazing how little mapping is taking place,” he says. “If we had a map of every city, we’d have a vision of what needs to be done.”

Unlike the gardeners at the corner of Lucerne and Balsam, most people wondering what might be lurking in their soil don’t have a team of environmental scientists standing by to help. Affordable soil testing is often limited to laboratories affiliated with the U.S. Department of Agriculture’s Cooperative Extension System, which measure nutrients, acidity, organic content, and occasionally lead or other metals—but rarely other potential contaminants.^18^ If they do, the costs add up quickly. For example, Pennsylvania State University’s College of Agricultural Sciences charges $65 to test one sample for cadmium, copper, lead, nickel, chromium, and zinc. Add arsenic, mercury, molybdenum, and selenium, and the price rises to $160. PCBs cost another $80.^19^ PAHs are not on Penn State’s menu, but elsewhere testing for the 16 PAHs regulated by the EPA costs $250, says Ganga Hettiarachchi, an environmental chemist at Kansas State University.

Yet testing a single sample is rarely sufficient because contaminants occur patchily, says Hettiarachchi, who is studying garden soil contaminants in seven cities and food crops’ absorption of them under various conditions. For instance, lead is often concentrated near foundations of old houses and surface runoff pathways in residential yards, but hot spots can turn up anywhere an old painted board was discarded, say, or a long-gone fruit tree was sprayed with lead-arsenate pesticides.^1^^,^^20^

Furthermore, a recent Brown University study showed that lead contamination can spread farther and penetrate deeper than expected. Soil data from Rhode Island yards showed that lead-based paint spread more than 400 feet from nearby water towers, and often penetrated more than 12 inches below the soil surface.^14^ “The heterogeneity of contaminant distribution is one of the biggest challenges,” says Hettiarachchi. “You cannot actually afford to run so many samples.”

Gardeners often wind up testing for lead only, if anything, which Heiger-Bernays says can serve as a sentinel signaling the presence of other contaminants. She recommends gardeners target their testing to areas most likely to be contaminated, such as near foundations or old painted structures, and they can keep costs down by combining several samples taken throughout a key planting area into a single sample for testing. Or, she says, skip the testing and just proceed as though the soil were contaminated.^18^

## Exposures and Health Impacts

Exposure to pollutants while gardening comes mainly from accidentally ingesting soil or inhaling contaminated dust, either while gardening or after tracking it home on clothing, shoes, and tools, according to interim guidelines for safe urban gardening from the EPA.^4^ The risk is greatest for small children, who not only are most vulnerable to toxicants but also gleefully put dirty fingers directly into their mouths.

Produce itself tends to be relatively safe, provided it wasn’t grown in heavily contaminated soil and is washed before eating.^4^ Most food crops tend not to absorb contaminants, and what little they do absorb generally stays in the roots.^4^^,^^21^ (One notable exception is rice, which absorbs arsenic unusually well.^22^) Certain contaminants, like zinc, kill plants before they reach concentrations dangerous to people, says Rufus Chaney, a research agronomist with the U.S. Department of Agriculture.

As urban agriculture flourishes and diversifies, however, at least one new exposure pathway has come to light: Health officials recently reported elevated lead levels in the edible portion of eggs from chickens raised in New York City community gardens.^23^ These chickens had been kept in areas with maximum soil lead concentrations of 600 ppm. The eggs were not likely to pose a health risk, the authors say, although eggs from chickens living on higher-lead soils possibly could. But overall, Chaney says, concerns focus on the ingestion of soil, not food.

Experts interviewed for this story could not recall a single case where illness had been traced directly to contaminated garden soil—a connection that in any case would be very difficult to prove. Yet for lead and other contaminants, garden soil may join other sources of exposure that add up for kids already at high risk, says Heiger-Bernays. “We know that urban centers like … Dorchester have these really recalcitrant elevated blood lead [levels] in some of the kids,” she says. “We figure that by adjusting some of the soil lead, we’ll be decreasing their overall exposure, because the lead in the soils ends up as lead in the dust in the home.”

Elevated blood lead levels in children are strongly linked with cognitive, motor, behavioral, and physical problems, including an increased risk of poor school performance and criminal behavior.^24^^,^^25^^,^^26^^,^^27^ A parallel body of research, much by Mielke and colleagues, shows a strong relationship between elevated soil lead and elevated blood lead in children.^13^^,^^28^^,^^29^^,^^30^^,^^31^^,^^32^ And while a 1998 pooled analysis of 12 studies found that lead-contaminated ﬂoor dust was a greater contributor to children’s blood lead levels than lead-contaminated soil, it nevertheless predicted a geometric mean blood lead level of 3.5 µg/dL in children living in homes with soil lead levels of 500 ppm when floor dust lead levels were very low.^33^ By comparison, the Centers for Disease Control and Prevention (CDC) currently considers 5 µg/dL the threshold for “elevated” blood lead, while pointing out that “no safe blood lead level in children has been identified.”^34^

But experts debate just how concerned gardeners should be about lead. The current EPA hazard standard of 400 ppm for bare soil in children’s play areas is generally viewed as the green light for gardening freely in unremediated soil.^35^ This standard is based on the EPA’s Integrated Exposure Uptake Biokinetic (IEUBK) model, which assesses the risk of elevated blood lead in a young child exposed to environmental lead from multiple sources. This model assumes that 30% of the lead in soil and dust ingested by children under age 7 is bioavailable—that is, it is absorbed into their bloodstream.^36^ But the IEUBK defines elevated blood lead as 10 µg/dL, twice the CDC’s threshold.

Individual states including Massachusetts, Minnesota, and California have established lower soil lead standards to protect children, and many European nations regulate soil lead at 100 ppm.^37^ (On average, the values that the EPA and other U.S. authorities use to regulate lead, cadmium, arsenic, nickel, chromium, mercury, copper, and zinc in soil are 10 times higher than elsewhere.^37^) “Four hundred [ppm] doesn’t cut it,” Heiger-Bernays says.

In a new document intended as a practical guide to safe urban gardening, she advises against gardening directly in soil with more than 200 ppm lead, and even recommends adding clean amendments to soil with more than 100 ppm lead.^18^ She arrived at those low action levels by balancing what she says is a strictly risk-based lead concentration of 2–50 ppm with consideration for what gardeners can realistically achieve. Even so, the levels are low enough to be “almost heretic” and are sure to get her lambasted by regulators, she says.

But Chaney says the EPA standard of 400 ppm is sufficiently protective for gardening. He points to his own unpublished research indicating that less than 5–10% of the lead in urban garden soil is bioavailable, compared with the 30% assumed by the IEUBK model.^38^ By contrast, the lead in unamended soil at contaminated mining sites can average an estimated 90% bioaccessibility.^39^

Garden soils may be safer than other urban soils because they receive regular additions of phosphorus through compost and other amendments, which speeds up the formation of pyromorphate, an insoluble compound of lead, say Hettiarachchi and Chaney. ^40^^,^^41^^,^^42^^,^^43^ In a forthcoming paper, Hettiarachchi and colleagues found that adding compost to soil reduced the estimated bioavailability of lead by 20–30%, compared with unamended soil.^3^ Chaney also points out that humans take up far less lead when they ingest it within a few hours of a meal than when they ingest it on an empty stomach.^44^^,^^45^

A considerable amount of research has gone into developing a cheap and easy test for lead bioavailability as part of a quest for a sure-fire way to improve soil safety by amending it, rather than replacing it.^42^ Yet for now such tests remain under development and confined to research laboratories, so there’s no way for a gardener to know for sure whether his or her high-lead soil might actually be fairly safe.

## Cleaning the Soil

The most thorough solution to cleaning up a garden is to remove the contaminated soil, then lay down a special fabric barrier topped with clean soil.^4^ But that’s a huge undertaking that can cost thousands of dollars, even for a small yard, putting it out of reach for most gardeners.^46^

Simply installing the barrier fabric and new soil on top of the old is a more feasible option. So is building raised beds filled with clean soil—especially for root crops—and covering any exposed contaminated soil with mulch or grass. Less problematic soils can be amended by mixing in plenty of compost to dilute contaminants and bind them to soil particles. Gardeners can further reduce their exposure by peeling root crops, removing the outer leaves of leafy crops, washing their produce and hands before eating, and leaving dirty garden gear outside.^4^

Although it can be tricky, ideally gardeners should also test incoming compost or soil because there’s little guarantee it will be much better than the old soil, says Heiger-Bernays. She and her students have found that few authorities either enforce rules governing what goes into compost or test the final product, although some voluntary standards do exist, such as the U.S. Composting Council’s Seal of Testing Assurance.^47^

Furthermore, contaminated compost is not as rare as a gardener might hope. For example, in 2011 Heiger-Bernays documented a spike in lead levels in Boston’s municipal compost to around 350 ppm. As a result, the city temporarily stopped delivering its cherished compost to Boston gardens. The cause of the spike was never confirmed, although sources speculate that old painted wood may have been tossed into the compost stream, or leaf blowers may have kicked up old paint particles around house foundations. (Boston’s new composting contractor, City Soil, appears to have resolved the problem.) Boston compost also had high levels of PAHs when the city added street sweepings to its mix, a practice it has since abandoned, says Heiger-Bernays. And since 2000, plant-killing compost has surfaced in more than a dozen states after the introduction of pyridine and pyrimidine carboxylic acids, persistent herbicides that do not break down during the composting process.^48^^,^^49^

To top it off, there is also some evidence that fresh, clean soil can pick up contamination from the garden site. For example, raised beds may become contaminated with high-lead soil blown in from the surrounding garden.^50^

Boston is a gardening hub, with around 175 community gardens in which some 3,500 families grow produce worth $1.5 million each year.^51^ The city spends around $300,000 annually to build new community gardens or renovate old ones. This figure is matched by private and foundation support through organizations such as Boston Natural Areas Network. Given that commitment, the city’s recent embrace of commercial farming as a way to bring employment, affordable produce, and an economic boost to the inner city seemed a natural step. New zoning regulations to make space for farms within city limits are slated for signing by the outgoing mayor, Thomas M. Menino, in December 2013.^52^

A provision in the new regulations specifically addressing soil contamination sets Boston apart from most other cities bitten by the urban ag bug.^53^ “Due to Boston’s industrial history and its archaeology and the oldness of the houses, there was always a burden of heavy metal concentrations in the soil. So we felt it was necessary that people farm wisely to protect not only themselves but anyone else from the toxic metals,” says Thomas Plant, director of special projects at the Boston Public Health Commission, which developed the soil contamination provision.

One vocal councilman wanted the city to require a professional environmental site assessment with extensive soil testing and replacement of all contaminated soil on city-owned lots used for farming. This costly requirement “would kill urban agriculture in the city of Boston,” says Plant. The final regulations give would-be farmers the more practical option of simply assuming the soil is polluted, covering it with barrier fabric, and trucking in clean soil to grow in. Most farmers are expected to take that route.

Soon after their September visit to the garden at Lucerne and Balsam, Heiger-Bernays’s students finished testing the soil samples. They were pleased to find that lead maxed out at 220 ppm, even near the old houses. Samples taken from a pile of the new city compost had low lead, too, at 120 ppm. Levels of arsenic and other metals were also low or nondetectable throughout the garden.

“That was a really nice surprise,” says Heiger-Bernays, who has identified lead levels up to 3,000 ppm in other community gardens bordered by lead-painted homes. She chalked up the healthy soil to Bleach and her fellow gardeners diligently applying compost, year after year. Further testing and research into the site’s history will tell more, but for now it seemed the renovators would need only to replace the soil at select spots and add more compost to keep the garden at Lucerne and Balsam safe for growing by any measure.
